# Supplementary material for: The incremental value of computed tomography of COVID-19 pneumonia in predicting ICU admission
Source: Sci Rep. 2021 Aug 2;11:15619. doi: 10.1038/s41598-021-95114-3 (PMC8329253; doi:10.1038/s41598-021-95114-3)
Supplement: Supplementary file 1 — Supplementary Information. [file 41598_2021_95114_MOESM1_ESM.pdf]

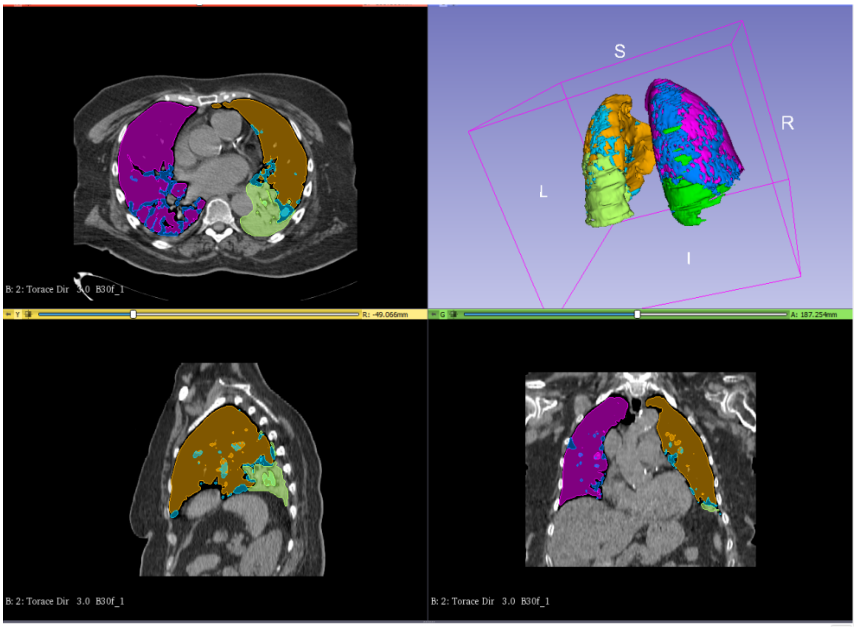

Suppl. Fig. 1 Example of segmentation of CT images. Well-Areated Lung (WAL) area are depicted in purple (right lung) and orange (left lung), Ground Glass Opacities (GGO) area are depicted in blue (right lung) and light blue (left lung) and Consolidation area (Consolid) are depicted in green (right lung) and light green (left lung).

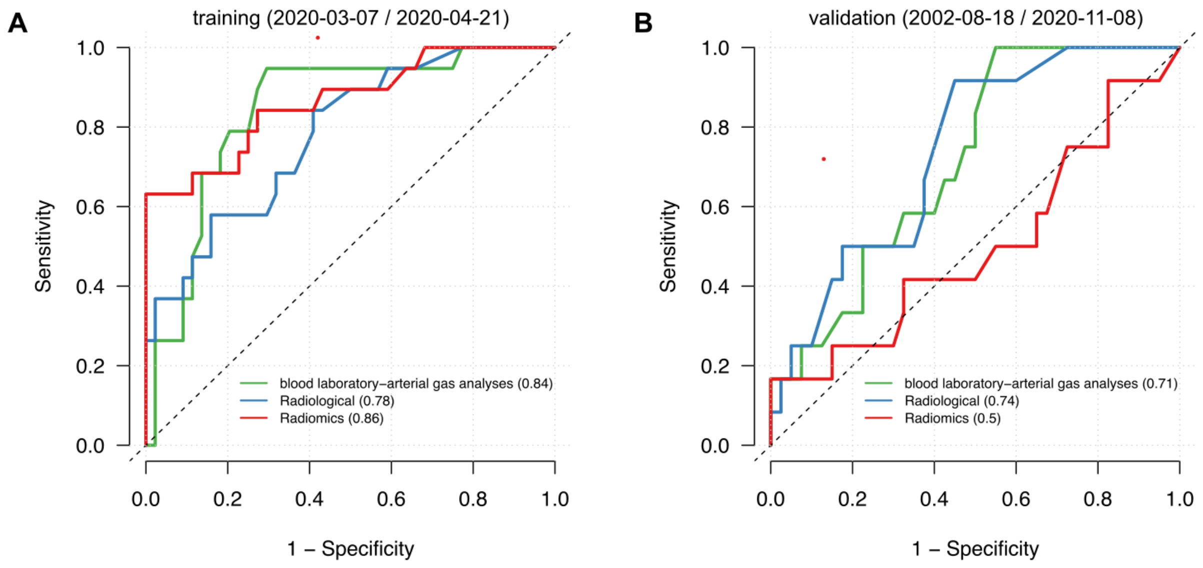

Supplementary Fig. 2. Performance of 3 simple models in predicting ICU admission. Receiving Operating Characteristic (ROC) curve analysis of the blood laboratory-arterial gas analyses features (green line), radiological features (sky blue line) and radiomics features (red line) in the training (A) and validation (B) sets. The values reported in parentheses refer to Area Under the ROC curves.

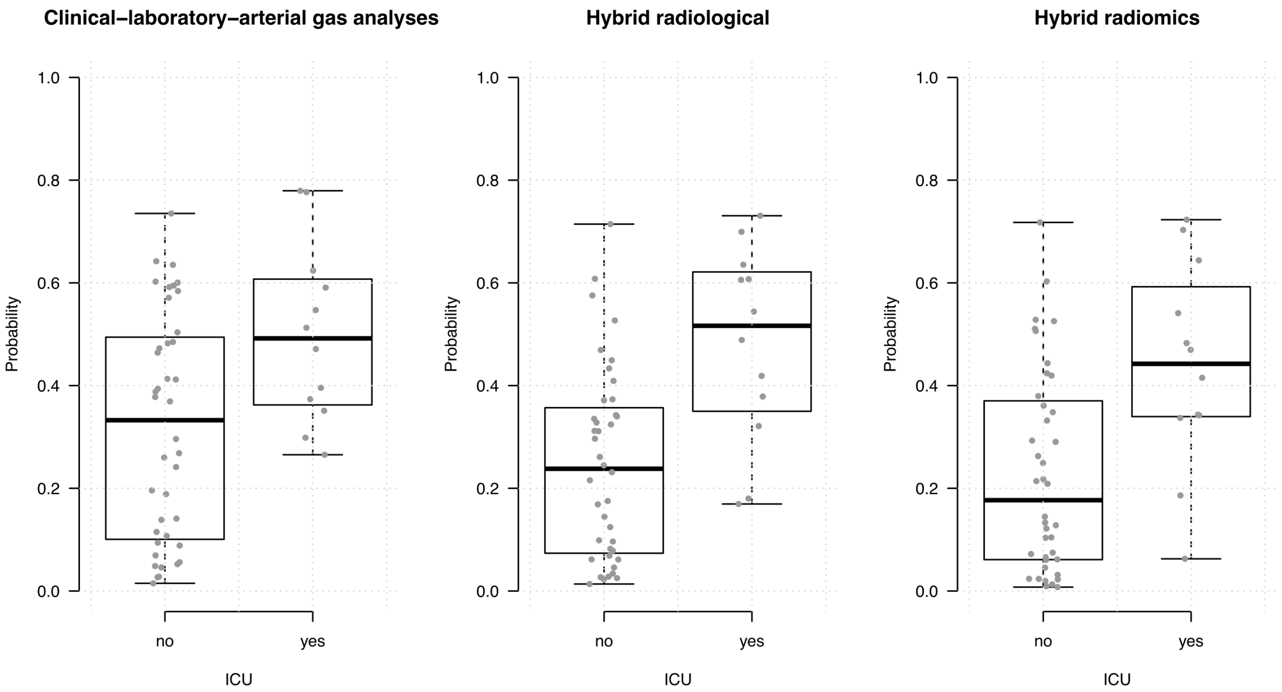

Supplementary Fig. 3. Box plots of the distribution of the probability of ICU admission estimated using the blood laboratory-arterial gas analyses (left), Hybrid radiological (middle) and Hybrid radiomics (right) models in the patients of the validation set who were not admitted (ICU=no) or required admission (ICU=yes) to ICU. No patient with estimated probability below 0.25 (laboratory-arterial gas analyses model), 0.15 (Hybrid radiology model) and 0.05 (Hybrid radiomic model) required ICU admission.

Supplementary Table 1. Radiomic features for image texture analysis extracted using the 3DSlicer software and the module radiomic.

Parameter type	Parameter name	Description	Selected in Clinical-laboratory-arterial gas analysis	Selected in Radiological	Selected in Radiomics	Selected in Hybrid radiological	Selected in Hybrid radiological + radiomic
Clinical-laboratory-arterial gas analysis	Age	years of age at admission to the Emergency Room	X			X	
Clinical-laboratory-arterial gas analysis	P/F	the Horowitz (P/F) Index is calculated as the ratio between the arterial partial pressure of oxygen [PaO2] measured(in mmHg) by blood gas analysis and fraction of inspired oxygen [FiO2]	X			X	X
Clinical-laboratory-arterial gas analysis	LDH	serum lactate dehydrogenase (UI/L)	X			X	X
Clinical-laboratory-arterial gas analysis	D-dimer	D-dimer (µg/mL FEU)					
Clinical-laboratory-arterial gas analysis	PCR	C-Reactive Protein (mg/dL)					
Clinical-laboratory-arterial gas analysis	Lymphocytes	Blood lymphocytes count (103/uL)					
Radiological	% Consolid	corresponds to % of the volume of areas of consolidated lung tissue after manually corrected software segmentation of chest CT images		X		X	X
Radiological	% Ground Glass	corresponds to % volume of areas of ground glass opacities in the lung after software segmentation of chest CT images					
Radiological	% Normal lung	corresponds to % of the volume of areas of normally areated lung tissue after software segmentation of chest CT images		X			
Radiomics	GrayLevelVariance	the variance in gray level intensities for the zones					
Radiomics	HighGrayLevelEmphasis	Measures the distribution of the higher gray-level values, with a higher value indicating a greater concentration of high gray-level values in the image.					
Radiomics	DependenceEntropy	Measures the uncertainty/randomness in dependence.					
Radiomics	DependenceNonUniformity	Measures the similarity of dependence throughout the image, with a lower value indicating more homogeneity among dependencies in the image.			X		
Radiomics	GrayLevelNonUniformity	Measures the similarity of gray-level intensity values in the image, where a lower GLN value correlates with a greater similarity in intensity values.					
Radiomics	SmallDependenceEmphasis	A measure of the distribution of small dependencies, with a greater value indicative of smaller dependence and less homogeneous textures.					
Radiomics	SmallDependenceHighGrayLevelEmphasis	Measures the joint distribution of small dependence with higher gray-level values.			X		

Radiomics	DependenceNonUniformityNormalized	Measures the similarity of dependence throughout the image, with a lower value indicating more homogeneity among dependencies in the image. This is the normalized version of the DLN formula.					
Radiomics	LargeDependenceEmphasis	A measure of the distribution of large dependencies, with a greater value indicative of larger dependence and more homogeneous textures.					
Radiomics	LargeDependenceLowGrayLevelEmphasis	Large Dependence Low Gray Level Emphasis (LDLGLE)			X		X
Radiomics	DependenceVariance	Measures the variance in dependence size in the image.					
Radiomics	LargeDependenceHighGrayLevelEmphasis	Measures the joint distribution of large dependence with higher gray-level values.					
Radiomics	SmallDependenceLowGrayLevelEmphasis	Measures the joint distribution of small dependence with lower gray-level values.					
Radiomics	LowGrayLevelEmphasis	Measures the distribution of low gray-level values, with a higher value indicating a greater concentration of low gray-level values in the image.					
Radiomics	JointAverage	Returns the mean gray level intensity of the distribution					
Radiomics	SumAverage	Measures the relationship between occurrences of pairs with lower intensity values and occurrences of pairs with higher intensity values.					
Radiomics	JointEntropy	A measure of the randomness/variability in neighborhood intensity values.					
Radiomics	ClusterShade	A measure of the skewness and uniformity of the GLCM. A higher cluster shade implies greater asymmetry about the mean.					
Radiomics	MaximumProbability	Indicates the occurrences of the most predominant pair of neighboring intensity values.					
Radiomics	Idmn	Inverse Difference Moment Normalized is a measure of the local homogeneity of an image.					
Radiomics	JointEnergy	A measure of homogeneous patterns in the image. A greater Energy implies that there are more instances of intensity value pairs in the image that neighbor each other at higher frequencies.					
Radiomics	Contrast	A measure of the local intensity variation, favoring values away from the diagonal i=j. A larger value correlates with a greater disparity in intensity values among neighboring voxels.					
Radiomics	DifferenceEntropy	A measure of the randomness/variability in neighborhood intensity value differences.					
Radiomics	InverseVariance	Assesses variations in intensity of voxels close to each other and therefore quantifies another aspect of homogeneity					

Radiomics	DifferenceVariance	A measure of heterogeneity that places higher weights on differing intensity level pairs that deviate more from the mean.					
Radiomics	Idn	Inverse Difference Normalized is another measure of the local homogeneity of an image. Unlike Homogeneity1, IDN normalizes the difference between the neighboring intensity values by dividing over the total number of discrete intensity values.					
Radiomics	Idm	Inverse Difference Moment is a measure of the local homogeneity of an image.					
Radiomics	Correlation	Is a value between 0 (uncorrelated) and 1 (perfectly correlated) showing the linear dependency of gray level values to their respective voxels in the GLCM.			X		
Radiomics	Autocorrelation	A measure of the magnitude of the fineness and coarseness of texture.					
Radiomics	SumEntropy	A sum of neighborhood intensity value differences.					
Radiomics	SumSquares	A measure in the distribution of neigboring intensity level pairs about the mean intensity level in the GLCM.					
Radiomics	ClusterProminence	Cluster Prominence is a measure of the skewness and asymmetry of the GLCM. A higher values implies more asymmetry about the mean while a lower value indicates a peak near the mean value and less variation about the mean.					
Radiomics	Imc2	Informational Measure of Correlation 2 assesses the correlation between the probability distributions of i and j (quantifying the complexity of the texture). The range of IMC2 = [0, 1), with 0 representing the case of 2 independent distributions (no mutual information) and the maximum value representing the case of 2 fully dependent and uniform distributions					
Radiomics	Imc1	Informational Measure of Correlation 1 assesses the correlation between the probability distributions of i and j (quantifying the complexity of the texture), using mutual information I(x, y). In the case where the distributions are independent, there is no mutual information and the result will therefore be 0. In the case of uniform distribution with complete dependence, mutual information will be equal to log2(Ng). Ng=matrix size					
Radiomics	DifferenceAverage	Measures the relationship between occurrences of pairs with similar intensity values and occurrences of pairs with differing intensity values.					

Radiomics	Id	Inverse Difference is a measure of the local homogeneity of an image. With more uniform gray levels, the denominator will remain low, resulting in a higher overall value.						
Radiomics	ClusterTendency	A measure of groupings of voxels with similar gray-level values.						
Radiomics	InterquartileRange	P75-P25; P25 and P75 are the 25th and 75th percentile of the image array, respectively			X			
Radiomics	Skewness	Measures the asymmetry of the distribution of values about the Mean value. Depending on where the tail is elongated and the mass of the distribution is concentrated, this value can be positive or negative.						
Radiomics	Uniformity	A measure of the sum of the squares of each intensity value. This is a measure of the homogeneity of the image array, where a greater uniformity implies a greater homogeneity or a smaller range of discrete intensity values.						
Radiomics	Median	The median gray level intensity within the ROI						
Radiomics	Energy	A measure of the magnitude of voxel values in an image. A larger values implies a greater sum of the squares of these values						
Radiomics	RobustMeanAbsoluteDeviation	The mean distance of all intensity values from the Mean Value calculated on the subset of image array with gray levels in between, or equal to the 10th and 90th percentile						
Radiomics	MeanAbsoluteDeviation	The mean distance of all intensity values from the Mean Value of the image array						
Radiomics	TotalEnergy	The value of Energy feature scaled by the volume of the voxel in cubic mm			X			
Radiomics	Maximum	Maximum gray level within the ROI						
Radiomics	RootMeanSquared	The square-root of the mean of all the squared intensity values. It is another measure of the magnitude of the image values.						
Radiomics	90Percentile	90th percentile of gray levels within the ROI						
Radiomics	Minimum	Minimum gray level within the ROI						
Radiomics	Entropy	Specifies the uncertainty/randomness in the image values. It measures the average amount of information required to encode the image values						
Radiomics	Range	Range of gray values in the ROI						
Radiomics	Variance	The mean of the squared distances of each intensity value from the Mean value. This is a measure of the spread of the distribution about the mean.						
Radiomics	10Percentile	10th percentile of gray levels within the ROI						

Radiomics	Kurtosis	A measure of the peakedness of the distribution of values in the image ROI. A higher kurtosis implies that the mass of the distribution is concentrated towards the tail(s) rather than towards the mean. A lower kurtosis implies the reverse: that the mass of the distribution is concentrated towards a spike near the Mean value					
Radiomics	Mean	The average gray level intensity within the ROI					
Radiomics	ShortRunLowGrayLevelEmphasis	Measures the joint distribution of shorter run lengths with lower gray-level values.					
Radiomics	LowGrayLevelRunEmphasis	Measures the distribution of low gray-level values, with a higher value indicating a greater concentration of low gray-level values in the image.					
Radiomics	GrayLevelNonUniformityNormalized	Measures the similarity of gray-level intensity values in the image, where a lower GLNN value correlates with a greater similarity in intensity values. This is the normalized version of the GLN formula.					
Radiomics	RunVariance	A measure of the variance in runs for the run lengths.			X		
Radiomics	LongRunEmphasis	A measure of the distribution of long run lengths, with a greater value indicative of longer run lengths and more coarse structural textures.					
Radiomics	ShortRunHighGrayLevelEmphasis	Measures the joint distribution of shorter run lengths with higher gray-level values.					
Radiomics	RunLengthNonUniformity	Measures the similarity of run lengths throughout the image, with a lower value indicating more homogeneity among run lengths in the image.					X
Radiomics	ShortRunEmphasis	A measure of the distribution of short run lengths, with a greater value indicative of shorter run lengths and more fine textural textures.					
Radiomics	LongRunHighGrayLevelEmphasis	Measures the joint distribution of long run lengths with higher gray-level values.					
Radiomics	RunPercentage	Measures the coarseness of the texture by taking the ratio of number of runs and number of voxels in the ROI. Values are in range Np<=RP<=1, with higher values indicating a larger portion of the ROI consists of short runs (indicates a more fine texture). Np be the number of voxels in the image					
Radiomics	LongRunLowGrayLevelEmphasis	Measures the joint distribution of long run lengths with lower gray-level values.					
Radiomics	RunEntropy	Measures the uncertainty/randomness in the distribution of run lengths and gray levels. A higher value indicates more heterogeneity in the texture patterns.					

Radiomics	HighGrayLevelRunEmphasis	Measures the distribution of the higher gray-level values, with a higher value indicating a greater concentration of high gray-level values in the image.					
Radiomics	RunLengthNonUniformityNormalized	Measures the similarity of run lengths throughout the image, with a lower value indicating more homogeneity among run lengths in the image. This is the normalized version of the RLN formula.					
Radiomics	ZoneVariance	Measures the variance in zone size volumes for the zones.					
Radiomics	SizeZoneNonUniformityNormalized	Measures the variability of size zone volumes throughout the image, with a lower value indicating more homogeneity among zone size volumes in the image. This is the normalized version of the SZN formula.					
Radiomics	SizeZoneNonUniformity	Measures the variability of size zone volumes in the image, with a lower value indicating more homogeneity in size zone volumes.					
Radiomics	LargeAreaEmphasis	A measure of the distribution of large area size zones, with a greater value indicative of more larger size zones and more coarse textures.					
Radiomics	SmallAreaHighGrayLevelEmphasis	Measures the proportion in the image of the joint distribution of smaller size zones with higher gray-level values.					
Radiomics	ZonePercentage	Measures the coarseness of the texture by taking the ratio of number of zones and number of voxels in the ROI.					
Radiomics	LargeAreaLowGrayLevelEmphasis	Measures the proportion in the image of the joint distribution of larger size zones with lower gray-level values.			X		
Radiomics	LargeAreaHighGrayLevelEmphasis	Measures the proportion in the image of the joint distribution of larger size zones with higher gray-level values.					
Radiomics	HighGrayLevelZoneEmphasis	Measures the distribution of the higher gray-level values, with a higher value indicating a greater proportion of higher gray-level values and size zones in the image.					
Radiomics	SmallAreaEmphasis	A measure of the distribution of small size zones, with a greater value indicative of more smaller size zones and more fine textures.					
Radiomics	LowGrayLevelZoneEmphasis	Measures the distribution of lower gray-level size zones, with a higher value indicating a greater proportion of lower gray-level values and size zones in the image.					X
Radiomics	ZoneEntropy	Measures the uncertainty/randomness in the distribution of zone sizes and gray levels. A higher value indicates more heterogeneity in the texture patterns.					

Radiomics	SmallAreaLowGrayLevelEmphasis	Measures the proportion in the image of the joint distribution of smaller size zones with lower gray-level values.			X			
Radiomics	Coarseness	A measure of average difference between the center voxel and its neighbourhood and is an indication of the spatial rate of change. A higher value indicates a lower spatial change rate and a locally more uniform texture.						
Radiomics	Complexity	An image is considered complex when there are many primitive components in the image, i.e. the image is non-uniform and there are many rapid changes in gray level intensity.						
Radiomics	Strength	Strength is a measure of the primitives in an image. Its value is high when the primitives are easily defined and visible, i.e. an image with slow change in intensity but more large coarse differences in gray level intensities.						
Radiomics	Busyness	A measure of the change from a pixel to its neighbour. A high value for busyness indicates a busy image, with rapid changes of intensity between pixels and its neighbourhood.						
